# Occupational Exposure to Petroleum-Derived Lubricants and Risk of Multiple Sclerosis

**DOI:** 10.1212/WNL.0000000000218718

**Published:** 2026-11-04

**Authors:** Anna Karin Hedström, Tomas Olsson, Lars Alfredsson

**Affiliations:** 1 Department of Clinical Neuroscience, Karolinska Institutet, Stockholm, Sweden;; 2 Institute of Environmental Medicine, Karolinska Institutet, Stockholm, Sweden; and; 3 Center for Occupational and Environmental Medicine, Region Stockholm, Sweden.

## Abstract

**Background and Objectives:**

Lung irritants such as smoking and organic solvents are established risk factors of multiple sclerosis (MS), with effects influenced by genetic susceptibility, particularly leukocyte antigen (HLA) DRB1*15:01 and HLA-A*02:01. Petroleum-derived lubricants represent another widespread occupational exposure with potential inflammatory properties, yet their contribution to MS etiology remains unclear. This study investigated associations between occupational exposure to petroleum-based lubricants and MS risk, including interactions with smoking and HLA genotypes.

**Methods:**

This investigation was based on a Swedish population-based case-control design. Incident MS cases were consecutively recruited by neurologists at 40 clinics (2005–2015). Inclusion criteria comprised residence in Sweden, age 16–70 years, and a diagnosis of MS confirmed by a neurologist according to the McDonald criteria. Controls were randomly selected from the national population register using density sampling and frequency-matched to cases by age, sex, and residential area. Exposure to petroleum-derived lubricants was assessed using questionnaire data. Logistic regression was used to estimate odds ratios (ORs) and 95% CIs for MS in relation to occupational exposure to petroleum-derived lubricants. Interactions with smoking, HLA-DRB1*15:01, and absence of HLA-A*02:01 were assessed on the additive scale using the attributable proportion due to interaction (AP).

**Results:**

The study population comprised 2,872 incident MS cases and 6,122 controls (mean age: 34.6 years; 72% female). Occupational exposure to petroleum-derived lubricants was associated with increased MS risk (OR 1.38, 95% CI 1.16–1.65), with a modest exposure-duration response. The association persisted when applying a 7-year exposure lag (OR 1.55, 95% CI 1.27–1.90). A positive interaction was identified between petroleum exposure and smoking (AP 0.34, 95% CI 0.10–0.55). Among the subset with available HLA data (2,070 cases and 2,899 controls), petroleum exposure interacted with the absence of HLA-A*02:01 (AP 0.32, 95% CI 0.05–0.59). A similar interaction was observed with HLA-DRB1*15:01 (AP 0.19, 95% CI -0.08-0.46), although this estimate did not reach statistical significance.

**Discussion:**

Occupational exposure to petroleum-derived lubricants was associated with MS risk, with stronger associations among smokers and participants with established genetic susceptibility. These findings add to the evidence that lung irritants and gene-environment interactions contribute to MS susceptibility but require replication in independent populations.

## Introduction

MS is a chronic, immune-mediated disorder of the CNS, resulting from a complex interplay between genetic and environmental factors.^1^ The most important genetic risk factors lie within the human leukocyte antigen (HLA) region. The HLA-DRB1*15:01 allele is associated with an approximately threefold increased risk of MS, whereas HLA-A*02:01 is associated with reduced risk.^2,3^ Established environmental risk factors include Epstein-Barr virus infection,^4,5^ limited sun exposure,^5^ adolescent obesity,^5^ and exposures to lung irritants such as cigarette smoke,^6^ organic solvents,^7^ and air pollution.^8^ The risk associated with these exposures is further elevated in the presence of HLA-DRB*15:01 and absence of HLA-A*02:01.^9-11^

Petroleum-derived lubricants are commonly encountered in industrial settings, where prolonged and recurrent exposure to aerosols, mists, and volatile compounds may provoke respiratory and systemic inflammation.^12-15^ Despite their biological plausibility as respiratory irritants, the potential role of petroleum-derived lubricants in MS susceptibility has not previously been investigated.

In a Swedish population-based case-control study, we investigated the association between occupational exposure to petroleum-based lubricants and the risk of MS and examined potential interactions between this exposure, smoking, HLA-DRB1*15:01, and absence of HLA-A*02:01.

## Methods

### Study Design and Study Participants

This study is part of the epidemiological investigation of multiple sclerosis (EIMS), a population-based case-control study involving the general Swedish population aged 16–70 years. Between April 2005 and December 2015, incident cases of MS were ascertained from 40 neurology departments across Sweden, including all university hospitals and most clinics involved in MS diagnosis and care. Since essentially all patients with suspected MS are referred to specialized neurology services within the tax-funded Swedish health care system, the study achieved nationwide coverage, with cases recruited from all Swedish counties. Diagnoses were made by a neurologist according to the McDonald criteria.^16,17^ For each case, 2 controls were randomly selected from the national population register by density sampling. Controls were frequency-matched at the group level according to age in 5-year strata, sex, and residential area. In total, 2,872 cases and 6,122 controls completed a standardized questionnaire on environmental exposures and lifestyle-related factors. Response rates were 93% among cases and 73% among controls. Participants who did not complete all questions were contacted by phone or email to obtain missing information. Blood samples were available from 2070 cases and 2,899 controls. Analyses of environmental exposures included the entire study population, and subanalyses of gene-environment interactions were conducted among participants with available genotyping data. Further details on the EIMS design and methodology have been published elsewhere.^18^

### Standard Protocol Approvals, Registrations, and Participant Consents

The study was approved by the Regional Ethical Review Board at Karolinska Institute (reference number 2004-252-1:4) and conducted in accordance with the ethical principles outlined in the Declaration of Helsinki. All participants provided written informed consent.

### Definition of Petroleum-Derived Oils and Lubricants

For each case, we estimated the index year as the year in which the first MS symptom occurred. Matched controls were assigned the same index year as their corresponding case.

Participants were asked to report occupational exposure separately for each of the following petroleum-derived lubricants: cutting lubricants and fluids, engine oil, hydraulic oil, and concrete form oil. For each reported exposure, individuals were further asked to provide information on the timing and average weekly duration. Exposure was defined as any occupational contact with one or more of these substances before the index year. Participants also provided information on all occupations held throughout working life, including occupational title, type of business or industry, and period of employment. Occupational information was used descriptively to characterize participants reporting exposure to petroleum-derived lubricants.

Smoking status was determined from self-reported information on past and current tobacco use. Participants who smoked at the index year were considered current smokers, while all others were classified as nonsmokers. This categorization is supported by previous evidence that the effects of smoking on MS risk, including its interactions with genetic factors, decrease gradually after smoking cessation.^18^

### Genotyping and Definition of Genetic Risk Factors

*HLA-DRB1* alleles were determined at two-field resolution. Genotyping was performed using the MS replication chip,^19^ and classical HLA alleles (*HLA-DRB1, HLA-A, HLA-B, HLA-DQA1*, and *HLA-DQB1*) were imputed with HLA*IMP:02.^20^ Participants were then classified according to the presence or absence of specific alleles (HLA-DRB1*15:01, DRB1*03:01, DRB1*08:01, DRB1*13:03, HLA-A*02:01, B*44:02, B*38:01, B*55:01, DQA1*01:01, DQB1*03:02, and DQB1*03:01).

### Statistical Analysis

We compared the odds of MS among participants reporting exposure to petroleum-derived lubricants with those of unexposed participants by fitting logistic regression models, both overall and stratified by sex. Unconditional logistic regression was used as the primary analytical approach to retain all available controls in the sex-stratified analyses. As a sensitivity analysis, we further performed conditional logistic regression based on the original matched case-control sets. Dose-response patterns were evaluated by including total duration of exposure to any petroleum-derived lubricant before the index year as a continuous variable in the logistic model. When participants reported exposure to multiple lubricant types during overlapping periods, overlapping years were counted only once. Inclusion of a quadratic term for exposure duration provided no evidence of a substantial departure from linearity (*p* = 0.10). To further characterize the exposure-response relationship, we performed additional analyses of exposure intensity and cumulative exposure among participants with available intensity data. Information on average weekly exposure duration was available for 173 exposed cases and 275 exposed controls. Exposure intensity was assessed as average hours of exposure per week, and cumulative exposure was defined as exposure duration multiplied by average weekly exposure intensity. We also examined each type of petroleum-derived lubricant separately.

To explore prespecified gene-environmental and lifestyle interactions, we calculated the attributable proportion (AP) due to interaction between exposure to petroleum-derived lubricants and each of the following: current smoking, presence of HLA-DRB1*15:01, and absence of HLA-A*02:01. The AP, defined as (OR_11_ – OR_10_ – OR_01_ + 1)/OR_11_, quantifies the excess risk attributable to the interaction beyond the sum of the individual effects, with values greater than zero suggesting that the exposures, given causality, act synergistically along a shared biological pathway.^21^ Confidence intervals for AP were derived using the delta method.^21^

Analyses were adjusted for design factors (age, sex, residential area), as well as ancestry (Nordic or non-Nordic), past infectious mononucleosis (yes, no, unknown), and adolescent body mass index (underweight, normal weight, overweight, or obese, as defined by the World Health Organization). Where appropriate, we further controlled for smoking and other HLA alleles independently associated with MS risk^2^: DRB1*03:01, DRB1*13:03, DRB1*08:01, B*44:02, B*38:01, B*55:01, DQA1*01:01, DQB1*03:02, and DQB1*03:01.

Additional lifestyle covariates including alcohol use, physical activity, and sun exposure were evaluated as potential confounders but were not included in the final models because they had negligible impact on the effect estimates. Alcohol consumption was categorized as none, low, moderate, or high consumption according to cutoffs used by Statistics Sweden. Participants reported their typical physical activity level during the previous year by selecting 1 of 4 predefined categories ranging from predominantly sedentary behavior to regular, sweat-inducing exercise at least 3 times per week. Sun exposure habits during the 5 years before inclusion, including current exposure, were assessed using 2 four-level items. Scores were summed to a total index ranging from 2 to 6, with higher values indicating greater exposure.

To address the possibility that exposure during a prodromal phase influenced the findings, we performed a sensitivity analysis applying a 7-year exposure lag. Participants were classified as exposed only if occupational exposure to petroleum-derived lubricants had occurred at least 7 years before the index year. Exposure occurring exclusively during the 7 years immediately preceding the index year was disregarded. The same covariate adjustment as in the primary analysis was applied. Educational level, shift work, and occupational exposure to organic solvents before the index year were evaluated in sensitivity analyses to assess potential residual confounding related to socioeconomic and occupational factors. Educational level was categorized as university or no university education. Shift work was defined as ever having worked between 9 pm and 7 am before the index year. Occupational exposure to organic solvents was defined as exposure to organic solvents, painting products, and varnish. All analyses were conducted using Statistical Analysis System version 9.4.

### Data Availability

Anonymized data underlying this article will be shared on reasonable request from any qualified investigator who wants to analyze questions that are related to the published article.

## Results

Our analysis included 2,872 cases and 6,122 controls matched by age, sex, and residential area. The mean age at onset was 34.6 years, and the median duration from the clinically overt disease onset to diagnosis was 1.0 year. Almost all cases were recruited within 1 year of diagnosis, and the questionnaires were completed a median of 2.0 years after disease onset. The characteristics of cases and controls, stratified by occupational exposure to petroleum-derived lubricants, are presented in Table 1. Men were more frequently exposed to petroleum-derived lubricants than women.

**Table 1:** Characteristics of Cases and Controls

	Total		Petroleum-derived oils and lubricants			
			Exposed		Nonexposed	
	Cases	Controls	Cases	Controls	Cases	Controls
N	2,872	6,122	251	399	2,621	5,723
Age at index (SD)	34.6 (10.6)	34.6 (10.7)	36.3 (9.9)	36.7 (10.8)	34.5 (10.7)	34.4 (10.7)
Male, n (%)	810 (28)	1,754 (29)	194 (77)	319 (80)	616 (24)	1,435 (25)
Female, n (%)	2062 (72)	4,368 (71)	57 (23)	80 (20)	2005 (76)	4,288 (75)
Nordic (n, %)	2,334 (81)	4,795 (78)	219 (87)	351 (88)	2,115 (81)	4,444 (78)
University education, n (%)	1,265 (44.0)	2,448 (40.0)	49 (19.5)	92 (23.1)	1,216 (46.4)	2,356 (41.2)
IM, n (%)	498 (18)	622 (10)	41 (17)	44 (11)	457 (18)	578 (10)
Never smoking, n (%)	1,366 (48)	3,533 (58)	98 (39)	222 (56)	1,267 (48)	3,311 (58)
Current smoking, n (%)	856 (30)	1,412 (23)	88 (35)	87 (22)	768 (29)	1,325 (23)
Past smoking, n (%)	650 (23)	1,177 (19)	64 (26)	90 (23)	586 (22)	1,087 (19)
Normal weight, n (%)	2,389 (83)	5,428 (89)	189 (75)	325 (81)	2,200 (84)	5,103 (89)
Overweight, n (%)	360 (13)	556 (9.1)	52 (21)	64 (16)	308 (12)	492 (8.6)
Obesity, n (%)	123 (4.3)	138 (2.3)	10 (4.0)	10 (2.5)	113 (4.3)	128 (2.2)
Physical activity score (SD)	2.6 (1.0)	2.7 (1.0)	2.6 (1.0)	2.6 (1.0)	2.6 (1.0)	2.7 (1.0)
Alcohol consumption, gram/wk (SD)	42.5 (62.8)	53.0 (89.2)	54.6 (60.8)	81.4 (133.3)	41.4 (62.8)	51.0 (84.9)
Sun exposure index (SD)	6.2 (1.8)	6.5 (1.9)	5.7 (1.8)	5.9 (1.9)	6.2 (1.8)	6.6 (1.9)
Shift work, n (%)	991 (34.5)	1918 (31.3)	129 (51.4)	210 (52.6)	862 (31.4)	1708 (29.9)
Organic solvents, n (%)	268 (9.3)	450 (7.4)	105 (41.8)	161 (40.4)	163 (6.2)	289 (5.0)
Exposure characteristics among participants exposed to petroleum-derived oils and lubricants						
Time since first exposure, y (SD)	—	—	15.4 (9.9)	14.9 (10.7)	—	—
Time since last exposure, y (SD)	—	—	6.5 (8.0)	5.7 (7.7)	—	—
Exposure duration, y (SD)	—	—	9.6 (9.1)	9.9 (8.8)	—	—
Exposure intensity, hours per week (SD)	—	—	13.7 (15.6)	10.8 (14.2)	—	—

Abbreviation: IM = infectious mononucleosis.

Exposure intensity was available for 173 exposed cases and 275 exposed controls.

Among participants reporting exposure to petroleum-derived lubricants, the most common occupational categories included metalworking (23%), construction (17%), automotive repair (11%), and machinery maintenance (10%). We observed a significant association between exposure to petroleum-derived lubricants and increased MS risk (OR 1.38, 95% CI 1.16–1.65) (Table 2). There was a significant dose-response pattern, with increased odds of MS with both increasing exposure duration (OR per year of exposure 1.02, 95% CI 1.01–1.03) and increasing exposure intensity (OR per additional hour of exposure per week 1.02, 95% CI 1.01–1.03). Similarly, increasing cumulative exposure was associated with increased odds of MS (OR per 10-unit increase 1.01, 95% CI 1.00–1.03). When exposure duration and exposure intensity were included simultaneously in the model, both variables remained independently associated with MS risk, with effect estimates similar to those observed in the separate models. In sex-stratified analyses, occupational exposure to petroleum-derived lubricants was associated with an increased risk of MS in both women (OR 1.46, 95% CI 1.03–2.07) and men (OR 1.40, 95% CI 1.14–1.73) (Table 2). When examining specific types of petroleum-derived lubricants separately, significant ORs were observed for all types but hydraulic oil (OR 1.23, 95% CI 0.98–1.55) (Table 3). Results from conditional logistic regression analyses remained consistent with those from the primary analyses (eTable 1).

**Table 2:** Associations Between Occupational Exposure to Petroleum-Derived Oils and Lubricants and Multiple Sclerosis Risk, Overall and Stratified by Sex

Exposure	Cases/controls	OR (95% CI)^a^	OR (95% CI)^a,c^
Petroleum-derived oils and lubricants			
No	2,621/5,723	1.0 (reference)	1.0 (reference)
Yes	251/399	1.43 (1.20–1.70)	1.38 (1.16–1.65)

Women			
Exposure	Cases/controls	OR (95% CI)^b^	OR (95% CI)^b,c^
Petroleum-derived oils and lubricants			
No	2005/4,288	1.0 (reference)	1.0 (reference)
Yes	57/80	1.48 (1.05–2.10)	1.46 (1.03–2.07)

Men			
Exposure	Cases/controls	OR (95% CI)^b^	OR (95% CI)^b-c^
Petroleum-derived oils and lubricants			
No	616/1,435	1.0 (reference)	1.0 (reference)
Yes	194/319	1.45 (1.18–1.78)	1.40 (1.14–1.73)

Abbreviation: OR = odds ratio.

a Adjusted for age, sex, residential area (according to study design).

b Adjusted for age and residential area (according to study design).

c Adjusted for ancestry, past infectious mononucleosis, body mass index at age 20, and smoking.

**Table 3:** Exposure to Specific Petroleum-Derived Oils and Lubricants and Risk of Multiple Sclerosis

Specific petroleum-derived oils and lubricants			
Exposure	Cases/controls	OR (95% CI)^a^	OR (95% CI)^a,b^
Cutting lubricants/fluids			
No	2,621/5,723	1.0 (reference)	1.0 (reference)
Yes	88/132	1.51 (1.14–2.00)	1.45 (1.09–1.93)
Engine oil			
No	2,621/5,723	1.0 (reference)	1.0 (reference)
Yes	146/248	1.34 (1.08–1.67)	1.30 (1.04–1.62)
Hydraulic oil			
No	2,621/5,723	1.0 (reference)	1.0 (reference)
Yes	135/244	1.27 (1.01–1.59)	1.23 (0.98–1.55)
Concrete form oil			
No	2,621/5,723	1.0 (reference)	1.0 (reference)
Yes	34/40	1.95 (1.22–3.11)	1.92 (1.20–3.07)

Abbreviation: OR = odds ratio.

a Adjusted for age, sex, residential area (according to study design).

b Adjusted for ancestry, past infectious mononucleosis, body mass index at age 20, and smoking.

The combination of smoking and petroleum-derived lubricants interacted to further elevate the risk of MS, with an AP of 0.34 (95% CI 0.10–0.55) (Table 4).

**Table 4:** Joint Associations of Smoking and Exposure to Petroleum-Derived Oils and Lubricants With Multiple Sclerosis Risk

Smoking	Petroleum-derived oils and lubricants	Cases/controls	OR (95% CI)^a^	OR (95% CI)^b^	AP (95% CI)
Nonsmoker	No	1853/4,398	1.0 (reference)		
Nonsmoker	Yes	163/312	1.29 (1.05–1.58)	1.24 (1.02–1.55)	
Current smoking	No	768/1,325	1.38 (1.25–1.54)	1.37 (1.23–1.52)	
Current smoking	Yes	88/87	2.49 (1.83–3.38)	2.50 (1.83–3.38)	0.34 (0.10–0.55)

OR = odds ratio.

a Adjusted for age, sex, residential area (according to study design), and ancestry.

b Adjusted for age, sex, residential area (according to study design), ancestry, past infectious mononucleosis, and body mass index.

Among those with available HLA data, the association between petroleum-derived lubricants and MS risk was similar to that observed in the full sample (adjusted OR 1.32, 95% CI 1.04–1.68). Petroleum-derived lubricants showed evidence of additive interaction with absence of HLA-A*02:01 (AP 0.32, 95% CI 0.05–0.59), while a similar but less precisely estimated pattern was observed for HLA-DRB1*15:01 (AP 0.19, 95% CI -0.08-0.46) (Tables 5 and 6).

**Table 5:** Joint Associations of HLA-DRB1*15:01 Status and Exposure to Petroleum-Derived Oils and Lubricants With Multiple Sclerosis Risk

HLA-DRB1*15:01	Petroleum-derived oils and lubricants	ca/co*	OR (95% CI)^a^	OR (95% CI)^b^	AP (95% CI)
Noncarrier	No	863/1969	1.0 (reference)	1.0 (reference)	
Noncarrier	Yes	80/122	1.44 (1.06–1.95)	1.38 (1.01–1.90)	
Carrier	No	1,034/754	3.18 (2.80–3.60)	4.04 (3.43–4.76)	
Carrier	Yes	93/54	4.44 (3.08–6.39)	5.44 (3.73–7.94)	0.19 (−0.08–0.46)

Abbreviation: AP = attributable proportion; OR = odds ratio.

a Adjusted for age, sex, residential area (according to study design), and ancestry.

b Adjusted for age, sex, residential area, ancestry, past infectious mononucleosis, smoking, body mass index, HLA-DRB1*03:01, DRB1*13:03, DRB1*08:01, A*02:01, B*44:02, B*38:01, B*55:01, DQA1*01:01, DQB1*03:02 and DQB1*03:01.

**Table 6:** Joint Associations of HLA-A*02:01 and Exposure to Petroleum-Derived Oils and Lubricants With Multiple Sclerosis Risk

HLA-A*02:01	Petroleum-derived oils and lubricants	ca/co*	OR (95% CI)^a^	OR (95% CI)^b^	AP (95% CI)
Carrier	No	789/1,465	1.0 (reference)	1.0 (reference)	
Carrier	Yes	70/103	1.23 (0.89–1.70)	1.15 (0.82–1.63)	
Noncarrier	No	1,108/1,258	1.65 (1.46–1.86)	1.60 (1.40–1.84)	
Noncarrier	Yes	103/73	2.57 (1.86–3.55)	2.58 (1.83–3.64)	0.32 (0.05–0.59)

Abbreviation: AP = attributable proportion; OR = odds ratio.

a Adjusted for age, sex, residential area (according to study design), and ancestry.

b Adjusted for age, residential area, ancestry, past infectious mononucleosis, smoking, body mass index, HLA-DRB1*15:01, DRB1*03:01, DRB1*13:03, DRB1*08:01, B*44:02, B*38:01, B*55:01, DQA1*01:01, DQB1*03:02 and DQB1*03:01.

Finally, we constructed combined exposure-genotype-smoking categories using nonsmokers unexposed to petroleum-derived lubricants and without either genetic risk factor as the reference. Compared with this reference group, HLA-DRB1*15:01–positive smokers without the HLA-A*02:01 allele and with no exposure to petroleum had an OR of 13.00 (95% CI 9.23–18.41); those with the same genetic profile who were exposed to petroleum-derived lubricants but did not smoke had an OR of 8.32 (4.38–15.82); and those with all 3 exposures (genetic risk profile, smoking, and petroleum-derived lubricants) had a 33.7-fold increased risk of MS (33.65, 95% CI 7.52–151). However, we note that some of these groups were small, resulting in reduced precision of the corresponding estimates (Table 7).

**Table 7:** OR and 95% CI for MS by Joint Categories of HLA-DRB1*15:01 Status, HLA-A*02:01 Status, Smoking, and Exposure to Petroleum-Derived Oils and Lubricants

DRB1*15:01	A*02:01	Smoking	Petroleum-derived oils/lubricants	ca/co*	OR (95% CI)^1^
−	+	−	−	238/782	1.0 (reference)
−	+	+	−	108/253	1.39 (1.05–1.82)
−	−	−	−	364/713	1.60 (1.31–1.95)
−	−	+	−	153/221	2.14 (1.65–2.77)
+	+	−	−	312/325	3.89 (3.07–4.93)
+	+	+	−	131/105	4.90 (3.59–6.68)
+	−	−	−	393/263	6.00 (4.72–7.64)
+	−	+	−	198/61	13.00 (9.23–18.41)
−	+	−	+	15/50	1.09 (0.68–1.64)
−	+	+	+	10/17	2.17 (0.96–4.90)
−	−	−	+	34/41	2.27 (1.38–3.76)
−	−	+	+	21/14	4.44 (2.18–9.04)
+	+	−	+	29/24	5.06 (2.79–9.16)
+	+	+	+	16/12	5.21 (2.34–11.1)
+	−	−	+	32/16	8.32 (4.38–15.82)
+	−	+	+	16/2	33.65 (7.52–151)

Abbreviation: OR = odds ratio.

The model was adjusted for age, sex, residential area (according to study design), ancestry, past infectious mononucleosis, body mass index, HLA-DRB1*03:01, DRB1*13:03, DRB1*08:01, B*44:02, B*38:01, B*55:01, DQA1*01:01, DQB1*03:02, and DQB1*03:01.

In the sensitivity analysis applying a 7-year exposure lag, 188 cases and 276 controls were classified as exposed. The association remained after disregarding exposure during the 7 years immediately preceding the index year (adjusted OR 1.55, 95% CI 1.27–1.90).

Additional analyses further adjusted for educational level, shift work, and occupational exposure to organic solvents yielded similar results (OR 1.32, 95% CI 1.08–1.62). Because occupational exposure to organic solvents was considered a particularly important potential confounder, we conducted several additional analyses to assess its influence. Mutual adjustment for petroleum-derived lubricants and organic solvents had negligible impact on the association between petroleum-derived lubricants and MS (OR 1.37, 95% CI 1.13–1.65). In the same model, occupational exposure to organic solvents also remained associated with increased odds of MS (OR 1.25, 95% CI 1.05–1.48). The association between petroleum-derived lubricants and MS was also preserved when the analysis was restricted to participants without occupational exposure to organic solvents (OR 1.46, 95% CI 1.16–1.83). When participants were classified according to exposure to petroleum-derived lubricants and organic solvents, elevated risks were observed in all exposure groups compared with participants unexposed to either agent (eTable 2).

## Discussion

We observed a significant association between occupational exposure to petroleum-derived lubricants and increased MS risk. The odds of MS rose modestly with increasing years of exposure, suggesting a dose-response relationship. It is important to note that the association was further amplified by smoking and by established genetic susceptibility profiles for MS.

Previous epidemiologic and experimental evidence supports a key role for lung irritants, including tobacco smoke, organic solvents, and ambient air pollution, in MS pathogenesis.^6-8^ Smoking, for example, has been consistently linked to MS in multiple populations, with compelling evidence of gene-environment interactions with HLA-DRB1*15:01 and HLA-A*02:01 status.^9^ Similarly, exposures to organic solvents have been shown to magnify MS risk, especially in genetically susceptible individuals.^11^ Our findings align with these observations, suggesting that occupational exposure to petroleum-derived lubricants, a prevalent source of fine aerosols, volatile compounds, and skin and respiratory irritants, may also trigger inflammatory and immune responses relevant to MS development.

Biologically, petroleum-derived lubricants generate airborne droplets and vapors that may penetrate deep into the lungs, resulting in local and systemic inflammation. Recent workplace and experimental studies show that these exposures possess a measurable oxidative potential, trigger proinflammatory cytokines, and may alter immune responses,^22-24^ potentially accelerating autoimmunity in predisposed individuals. Whether the increased MS risk associated with exposure to petroleum-derived lubricants is driven by inhalational exposure to oil mists, dermal absorption of specific additives, or a more general proinflammatory response is not fully understood. However, the observed interaction with smoking supports the hypothesis that airway irritation and inflammation could contribute to MS pathogenesis. Chronic irritation may promote local immune activation, either by expanding autoreactive T-cell populations or by inducing post-translational modifications of self-proteins that enhance their immunogenicity and resemblance to CNS antigens.^9^ These activated T cells, or newly generated neoantigens, could subsequently enter the circulation and penetrate the CNS.^25^ The observed interaction between exposure to petroleum-derived lubricants and smoking underscores the synergistic effects of multiple lung irritants in this process.

Our results suggest that individuals carrying HLA-DRB1*15:01 without HLA-A*02:01 may be particularly susceptible to the effects of petroleum-derived lubricants. This pattern is consistent with the well-documented interactions between lung irritants and genetic susceptibility in MS.^9-11^ Exposure to lung irritants can promote local inflammation and activate autoimmune T cells in the respiratory tract.^9^ In HLA-DRB1*15:01 carriers, the resulting class II molecules may present these self-peptides more efficiently, facilitating the expansion of pathogenic T-cell clones that can subsequently migrate to the CNS and contribute to MS.^25^ In contrast, HLA-A*02:01 has been associated with reduced chronic immune stimulation, potentially reflecting more effective control of persistent viral infections such as Epstein-Barr virus (EBV).^6,26,27^ This is further supported by recent work showing that MS-protective HLA class I alleles are linked to modulation of interferon signaling pathways.^28^ Its absence could, therefore, contribute to higher viral persistence and heightened immune activation, rendering individuals more vulnerable to inflammatory lung exposures and CNS-directed autoimmunity.

Several limitations of this study should be acknowledged. The exposure assessment relied on self-reported occupational histories, which introduces the possibility of recall bias and exposure misclassification. Although the use of detailed a questionnaire and the recruitment of incident cases likely reduced these risks, they cannot be entirely excluded. We also lacked detailed information on the formulation, composition, and refining processes of specific lubricant products and were, therefore, unable to assess whether particular constituents or changes in product composition over time influenced the observed associations. Such nondifferential misclassification would likely bias estimates toward the null. However, occupational titles reported by those indicating exposure were consistent with expected use of petroleum-based lubricants, and no major differences in professions or work sectors were observed between exposed cases and controls, suggesting similar reporting patterns.

Selection bias is another consideration. However, Sweden's health care system ensures equal access to care, and nearly all MS cases are referred to neurologic units. Although participation rates were higher among cases than controls, the response rate among controls was high for a population-based study. Furthermore, the smoking prevalence among controls was similar to that observed in the general population in the corresponding age groups,^29^ suggesting that major selection bias is unlikely. No significant differences in age, sex, smoking status, or exposure to petroleum-based lubricants were observed between participants with and without genetic data.

Although reverse causation is unlikely given the use of newly diagnosed cases, it cannot be completely ruled out. In addition, despite adjustment for known confounders, the possibility of residual confounding cannot be excluded. Occupational exposure to petroleum-derived lubricants may be correlated with other workplace exposures and socioeconomic factors that were not fully captured in this study. Nevertheless, additional adjustment for educational level and occupational exposure to organic solvents before the index year had negligible impact on the observed association. Despite the large sample size, some subgroups, particularly those with combined genetic and exposure profiles, were limited by small numbers, resulting in wide confidence intervals and reduced precision. Since EIMS has been used to examine several environmental and occupational exposures, the cumulative number of analyses increases the possibility that some findings may have arisen by chance. These findings should, therefore, be interpreted cautiously and require replication in independent populations. In conclusion, we provide new evidence that occupational exposure to petroleum-derived lubricants is associated with an increased risk of MS and that this risk is further augmented in smokers and individuals with HLA-DRB1*15:01 and absence of HLA-A*02:01. These findings support the broader hypothesis that inhaled lung irritants contribute to MS pathogenesis, especially in genetically susceptible individuals.

## Glossary

AP: attributable proportion
EIMS: epidemiological investigation of multiple sclerosis
HLA: human leukocyte antigen
MS: multiple sclerosis
ORs: odds ratios

## References

[1] Olsson T, Barcellos LF, Alfredsson L. Interactions between genetic, lifestyle and environmental risk factors for multiple sclerosis. Nat Rev Neurol. 2017;13(1):25-36. doi:10.1038/nrneurol.2016.187 27934854

[2] International Multiple Sclerosis Genetics ConsortiumWellcome Trust Case Control Consortium 2, Sawcer S, et al. Genetic risk and a primary role for cell-mediated immune mechanisms in multiple sclerosis. Nature. 2011;476(7359):214-219. doi:10.1038/nature10251 PMC3182531 21833088

[3] Moutsianas L, Jostins L, Beecham AH, et al. Class II HLA interactions modulate genetic risk for multiple sclerosis. Nat Genet. 2015;47(10):1107-1113. doi:10.1038/ng.3395 PMC4874245 26343388

[4] Bjornevik K, Cortese M, Healy BC, et al. Longitudinal analysis reveals high prevalence of Epstein-Barr virus associated with multiple sclerosis. Science. 2022;375(6578):296-301. doi:10.1126/science.abj8222 35025605

[5] Hedström AK. Risk factors for multiple sclerosis in the context of Epstein-Barr virus infection. Front Immunol. 2023;14:1212676. doi:10.3389/fimmu.2023.1212676 PMC10406387 37554326

[6] Rosso M, Chitnis T. Association between cigarette smoking and multiple sclerosis: a review. JAMA Neurol. 2020;77(2):245-253. doi:10.1001/jamaneurol.2019.4271 31841592

[7] Gerhardsson L, Hou L, Pettersson K. Work-related exposure to organic solvents and the risk of multiple sclerosis – a systematic review. Int Arch Occup Environ Health. 2021;94(2):221-229. doi:10.1007/s00420-020-01564-z PMC7872988 32880046

[8] Abbaszadeh S, Tabary M, Aryannejad A, et al. Air pollution and multiple sclerosis: a comprehensive review. Neurol Sci. 2021;42(10):4063-4072. doi:10.1007/s10072-021-05508-4 34341860

[9] Hedström AK. Smoking and its interaction with genetics in MS etiology. Mult Scler. 2019;25(2):180-186. doi:10.1177/1352458518801727 30251921

[10] Hedström AK, Segersson D, Hillert J, et al. Association between exposure to combustion-related air pollution and multiple sclerosis risk. Int J Epidemiol. 2023;52(3):703-714. doi:10.1093/ije/dyac234 PMC10244060 36629499

[11] Hedström AK, Hössjer O, Katsoulis M, Kockum I, Olsson T, Alfredsson L. Organic solvents and MS susceptibility: interaction with MS risk HLA genes. Neurology. 2018;91(5):e455-e462. doi:10.1212/WNL.0000000000005906 PMC6093765 29970406

[12] Massin N, Bohadana AB, Wild P, Goutet P, Kirstetter H, Toamain JP. Airway responsiveness, respiratory symptoms, and exposures to soluble oil mist in mechanical workers. Occup Environ Med. 1996;53(11):748-752. doi:10.1136/oem.53.11.748 PMC1128592 9038798

[13] Svendsen K, Hilt B. Exposure to mineral oil mist and respiratory symptoms in marine engineers. Am J Ind Med. 1997;32(1):84-89. doi:10.1002/(sici)1097-0274(199707)32:1<84::aid-ajim10>3.0.co;2-2 9131215

[14] Ameille J, Wild P, Choudat D, et al. Respiratory symptoms, ventilatory impairment, and bronchial reactivity in oil mist-exposed automobile workers. Am J Ind Med. 1995;27(2):247-256. doi:10.1002/ajim.4700270209 7755014

[15] Piacitelli GM, Sieber WK, O'Brien DM, Hughes RT, Glaser RA, Catalano JD. Metalworking fluid exposures in small machine shops: an overview. AIHAJ. 2001;62(3):356-370. doi:10.1080/15298660108984638 11434442

[16] Polman CH, Reingold SC, Edan G, et al. Diagnostic criteria for multiple sclerosis: 2005 revisions to the “McDonald Criteria”. Ann Neurol. 2005;58(6):840-846. doi:10.1002/ana.20703 16283615

[17] Polman CH, Reingold SC, Banwell B, et al. Diagnostic criteria for multiple sclerosis: 2010 revisions to the McDonald Criteria. Ann Neurol. 2011;69(2):292-302. doi:10.1002/ana.22366 PMC3084507 21387374

[18] Hedström AK, Hillert J, Olsson T, Alfredsson L. Smoking and multiple sclerosis susceptibility. Eur J Epidemiol. 2013;28(11):867-874. doi:10.1007/s10654-013-9853-4 PMC3898140 24146047

[19] International Multiple Sclerosis Genetics Consortium. Multiple sclerosis Genomic map implicates peripheral immune cells and Microglia in susceptibility. Science. 2019;365(6460):eaav7188. doi:10.1126/science.aav7188 PMC7241648 31604244

[20] Dilthey A, Leslie S, Moutsianas L, et al. Multi-population classical HLA type imputation. PLoS Comput Biol. 2013;9(2):e1002877. doi:10.1371/journal.pcbi.1002877 PMC3572961 23459081

[21] Andersson T, Alfredsson L, Källberg H, Zdravkovic S, Ahlbom A. Calculating measures of biological interaction. Eur J Epidemiol. 2005;20(7):575-579. doi:10.1007/s10654-005-7835-x 16119429

[22] Sauvain JJ, Suarez G, Hopf NB, et al. Oxidative potential of aerosolized metalworking fluids in occupational settings. Int J Hyg Environ Health. 2021;235:113775. doi:10.1016/j.ijheh.2021.113775 34058621

[23] Weatherly LM, Shane HL, Baur R, et al. Biological effects of inhaled crude oil. Toxicol Appl Pharmacol. 2022;449:116100. doi:10.1016/j.taap.2022.116100 35671832

[24] Gandomani EA, Mosaffa N, Zendehdel R, Kohneshahri MH, Vahabi M, Sabour S. Release of interleukin-1B evaluation among mineral oil mist-exposed workers. Toxicol Ind Health. 2022;38(5):270-276. doi:10.1177/07482337221090708 35465785

[25] Odoardi F, Sie C, Streyl K, et al. T cells become licensed in the lung to enter the central nervous system. Nature. 2012;488(7413):675-679. doi:10.1038/nature11337 22914092

[26] Lee SP, Thomas WA, Murray RJ, et al. HLA A2.1-restricted cytotoxic T cells recognizing a range of Epstein-Barr virus isolates through a defined epitope in latent membrane protein LMP2. J Virol. 1993;67(12):7428-7435. doi:10.1128/JVI.67.12.7428-7435.1993 PMC238208 7693972

[27] Agostini S, Mancuso R, Guerini FR, et al. HLA alleles modulate EBV viral load in multiple sclerosis. J Transl Med. 2018;16(1):80. doi:10.1186/s12967-018-1450-6 PMC5870171 29587799

[28] Lundtoft C, Pucholt P, Imgenberg-Kreuz J, et al. Function of multiple sclerosis-protective HLA class I alleles revealed by genome-wide protein-quantitative trait loci mapping of interferon signalling. PLoS Genet. 2020;16(10):e1009199. doi:10.1371/journal.pgen.1009199 PMC7644105 33104735

[29] Internet-based information. Accessed June 20, 2025. scb.se

